# Photoreduction of *Shewanella oneidensis* Extracellular Cytochromes by Organic Chromophores and Dye‐Sensitized TiO_2_

**DOI:** 10.1002/cbic.201600339

**Published:** 2016-11-08

**Authors:** Emma V. Ainsworth, Colin W. J. Lockwood, Gaye F. White, Ee Taek Hwang, Tsubasa Sakai, Manuela A. Gross, David J. Richardson, Thomas A. Clarke, Lars J. C. Jeuken, Erwin Reisner, Julea N. Butt

**Affiliations:** ^1^ School of ChemistryUniversity of East AngliaNorwich Research ParkNorfolkNR4 7TJUK; ^2^ School of Biological SciencesUniversity of East AngliaNorwich Research ParkNorfolkNR4 7TJUK; ^3^ School of Biomedical SciencesUniversity of LeedsLeedsLS2 9JTUK; ^4^ Department of ChemistryUniversity of CambridgeLensfield RoadCambridgeCB2 1EWUK; ^5^ Present address: Suntory Foundation for Life SciencesKyoto619-0284Japan

**Keywords:** cytochromes, electron transfer, heme proteins, photocatalysis, photoreduction

## Abstract

The transfer of photoenergized electrons from extracellular photosensitizers across a bacterial cell envelope to drive intracellular chemical transformations represents an attractive way to harness nature's catalytic machinery for solar‐assisted chemical synthesis. In *Shewanella oneidensis* MR‐1 (MR‐1), trans‐outer‐membrane electron transfer is performed by the extracellular cytochromes MtrC and OmcA acting together with the outer‐membrane‐spanning porin**⋅**cytochrome complex (MtrAB). Here we demonstrate photoreduction of solutions of MtrC, OmcA, and the MtrCAB complex by soluble photosensitizers: namely, eosin Y, fluorescein, proflavine, flavin, and adenine dinucleotide, as well as by riboflavin and flavin mononucleotide, two compounds secreted by MR‐1. We show photoreduction of MtrC and OmcA adsorbed on Ru^II^‐dye‐sensitized TiO_2_ nanoparticles and that these protein‐coated particles perform photocatalytic reduction of solutions of MtrC, OmcA, and MtrCAB. These findings provide a framework for informed development of strategies for using the outer‐membrane‐associated cytochromes of MR‐1 for solar‐driven microbial synthesis in natural and engineered bacteria.

## Introduction

Certain species of bacteria conduct electrons across the cell envelope in a manner that couples intra‐ and extracellular redox reactions. In nature, this extracellular electron transfer (EET) offers a competitive advantage in anaerobic habitats because electrons released by intracellular energy‐conserving pathways cross the cell membrane to reduce extracellular terminal electron acceptors that include particles containing Fe^III^ and Mn^IV^.^1^ However, EET can also exchange electrons between electrodes and bacteria known as electrotrophs. Electricity is generated when respiratory electrons from the oxidation of waste‐water‐derived electron donors are delivered to the anode of a microbial fuel cell.^2^ Microbial electrosynthesis is performed when the pathway of this respiratory electron transfer is reversed such that cathode‐derived electrons are delivered to intracellular enzymes.2b, 2c, 3 This approach affords strategies for tapping into the catalytic diversity and selectivity of enzymes for sustainable molecular syntheses that extend beyond H_2_ production and CO_2_ reduction while at the same time negating the need for time‐consuming and costly enzyme purification. Indeed, the prospect of using EET to couple robust and efficient extracellular light‐harvesting systems to intracellular catalysis represents a particularly attractive approach to sustainable solar‐assisted production of chemicals.3a, 4

**Figure 1:**
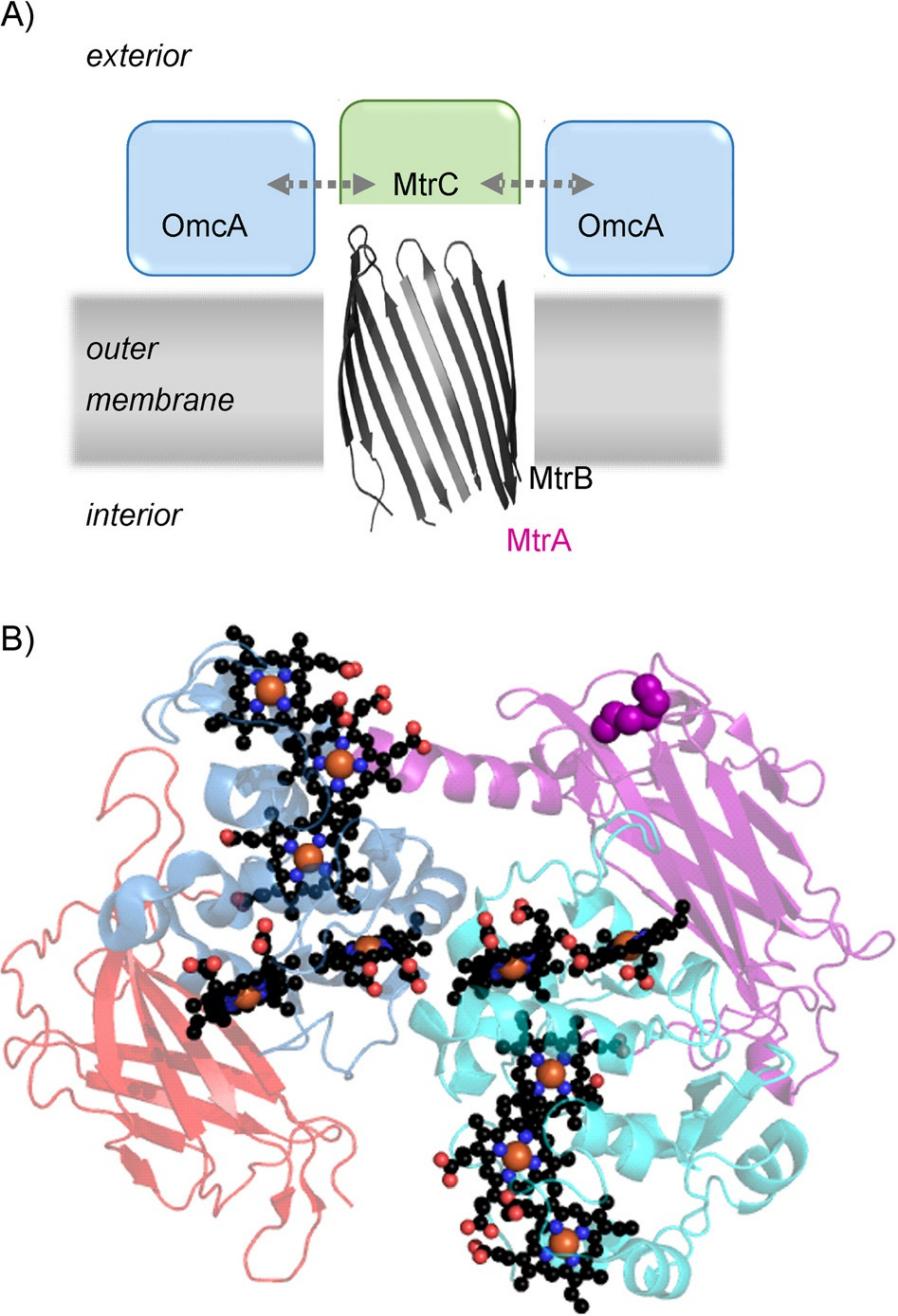
Proteins from *S. oneidensis* MR‐1 with key roles in extracellular electron transfer (EET). A) Schematic representation of the MR‐1 outer membrane illustrating the proposed locations and interactions of porin MtrB and the decaheme cytochromes MtrA, MtrC, and OmcA. The arrows indicate proposed interactions leading to electron exchange between MtrC and OmcA; see text for details. B) Structure of MtrC. Domains II (blue) and IV (cyan) contain heme groups shown as spheres with C in black, O in red, N in blue, and Fe in orange. Cysteine residues linked by disulfide bonds are shown as spheres in Domain III (purple). Structure rendered in PyMol from PDB ID: 4LM8.8b

*Shewanella oneidensis* MR‐1 (MR‐1) provides a model for the biochemistry and biophysics of EET^5^ and as a consequence a platform for the rational design of strategies for microbial electro‐ and photosynthesis. EET in this Gram‐negative electrotroph is underpinned by arrays of closely packed, protein‐bound heme cofactors that conduct electrons within and between proteins. The MR‐1 outer membrane is spanned by porin**⋅**cytochrome complexes that conduct electrons between the periplasm and external materials. Foremost amongst these is a tight 1:1 complex of two proteins: MtrA and MtrB (MtrAB, Figure 1 A). It is proposed that the decaheme cytochrome MtrA conducts electrons across the outer membrane by virtue of its insertion within a porin formed by MtrB.^6^ MtrAB forms a tight complex with the extracellular cytochrome MtrC, with which it exchanges electrons (Figure 1 A). During EET a second extracellular cytochrome, OmcA, can bind to, and exchange electrons with, MtrC.^7^ X‐ray diffraction has shown that MtrC and OmcA are structural homologues^8^ with ten heme groups bound in a staggered cross constellation (Figure 1 B). In addition, MtrC and OmcA were shown to possess one and two disulfide bonds, respectively. In vitro reduction of these bonds triggered tight binding of flavin mononucleotide (FMN) or riboflavin (RF) to both proteins;8b this might be significant because cellular studies have suggested that MtrC and OmcA act as flavocytochromes during anaerobic respiration.^9^

Several mechanisms by which MtrC and OmcA might facilitate electron exchange between the MR‐1 cell surface and extracellular materials have been proposed. Direct electron exchange might occur between these materials and the cofactors of MtrC and OmcA.^9^, ^10^ Electrons might be shuttled between these materials and the cell‐surface cytochromes through the diffusion of extracellular, redox‐active mediators that include flavins and low‐molecular‐weight Fe complexes.^11^ In addition, outer‐membrane extensions, coated with MtrC and OmcA and sometimes termed nanowires, have been implicated in mechanisms for electron exchange with remote materials across distances exceeding the cell dimensions.^12^

**Figure 2:**
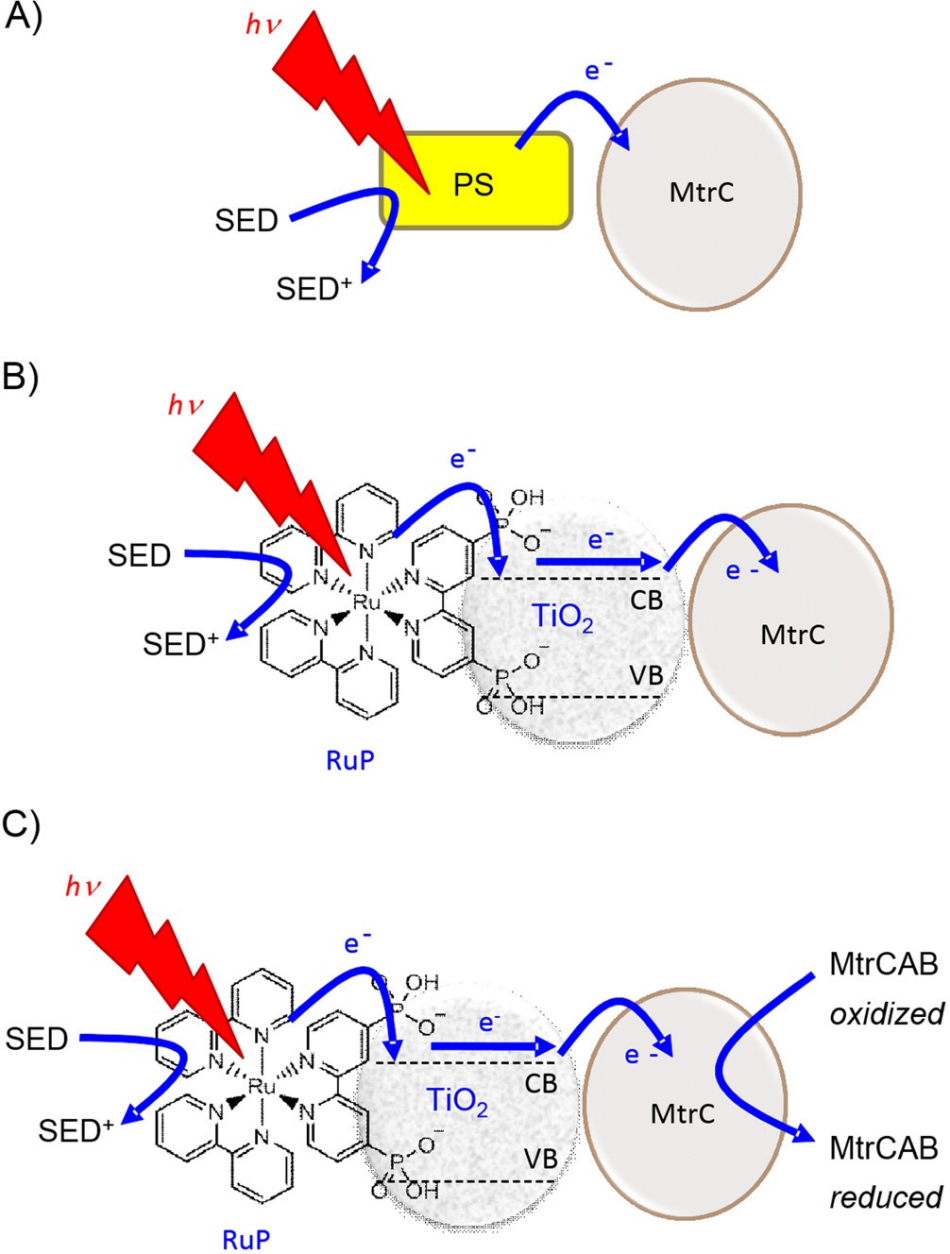
Schematic representation of strategies for photoreduction of MR‐1 cytochromes investigated in this work, illustrated for MtrC and MtrCAB. A) Photoreduction of MtrC by a soluble photosensitizer (PS), which may be an organic dye or an inorganic complex. B) Photoreduction of MtrC adsorbed on TiO_2_ nanoparticles sensitized with a phosphonated Ru^II^ dye (RuP). C) Photocatalytic reduction of solutions of MtrCAB by MtrC‐coated, dye‐sensitized TiO_2_ particles. SED: sacrificial electron donor. CB: conduction band. VB: valence band.

We have explored strategies to effect the photoreduction of MR‐1 extracellular cytochromes with the aid of water‐compatible light‐harvesting systems, because we envisage this as a route by which to facilitate solar‐assisted microbial production of chemicals by delivering photoexcited electrons to intracellular enzymes. We recently described how a monolayer of MtrC supported light‐driven charge transport to an underlying ultraflat gold electrode when coated with 3,4‐dihydroxybenzoic‐acid‐capped TiO_2_ nanocrystals (diameter ≈7 nm) that had been photosensitized with a phosphonated Ru^II^‐tris(bipyridine) dye.^13^ Here we report the photoreduction of solutions of MtrC, OmcA, and the MtrCAB complex by both biotic and abiotic photosensitizers that include organic dyes and transition‐metal complexes (Figure 2 A). We demonstrate photoreduction of MtrC and OmcA adsorbed on widely available TiO_2_ nanoparticles sensitized with a Ru dye (Figure 2 B). In addition, we show that MtrC or OmcA adsorbed on TiO_2_ particles serve as electron relays in the photoreduction of solutions of MtrC, OmcA, and the MtrCAB complex (Figure 2 C). These results extend the framework from which informed approaches to artificial microbial photosynthesis can be developed for strains of native and engineered^14^ bacteria that support EET through the action of multiheme cytochromes from, or homologous to those of, MR‐1.

## Results and Discussion

### Photoreduction of MtrC, OmcA, and MtrCAB by soluble photosensitizers

Evidence for visible‐light‐driven reduction of MtrC by soluble photosensitizers (Figure 2 A) was sought through electronic absorbance spectroscopy. Oxidized (ferric) heme groups contribute a single broad feature to spectra between 500 and 600 nm whereas two sharper and more intense features with maxima at 523 and 552 nm are indicative of reduced (ferrous) heme groups.^15^ Experiments were performed with eight photosensitizers, the structures and key photochemical properties of which are provided in Table S1 in the Supporting Information. FMN, RF, and flavin adenine dinucleotide (FAD) are naturally occurring flavins. The first two are secreted by MR‐1 and participate in EET.11a–11d Proflavine, fluorescein, and eosin Y are well‐studied light‐harvesting analogues of redox‐active molecules that serve as electron shuttles to enhance the performance of microbial fuel cells.^16^ [Ru(2,2′‐bpy)_3_]Cl_2_ (bpy=2,2′‐bipyridine) and [Ru(bpy)_2_{4,4′‐(PO_3_H_2_)_2_bpy}]Br_2_ (RuP) are robust light‐harvesting analogues of Fe^III^ chelates used as extracellular terminal electron acceptors by MR‐1.1a, 1b, 5a Triethanolamine (TEOA) and HEPES were included unless stated otherwise because these tertiary amines can serve as pH buffers and sacrificial electron donors during photoreduction.^17^

**Figure 3:**
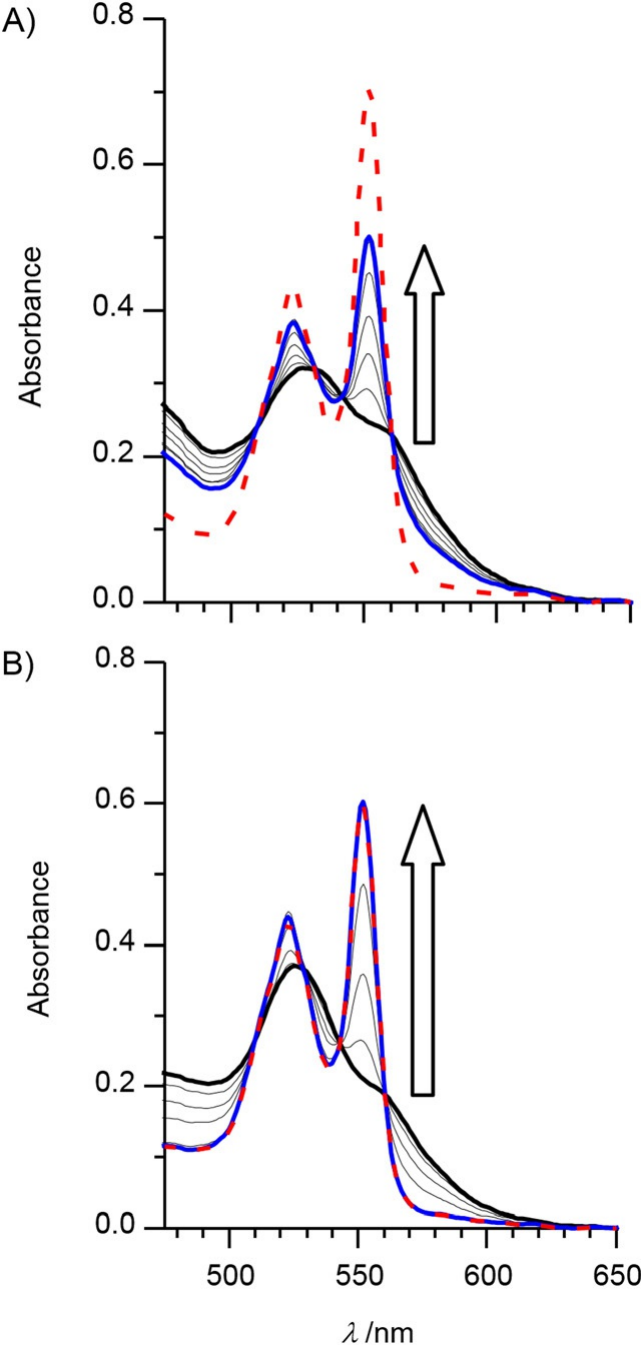
Photoreduction of MtrC by A) FMN, and B) eosin Y, visualized by electronic absorbance spectroscopy. A) Spectra of an MtrC (32 μm)/FMN (10 μm) solution as prepared (black line) and after illumination for 5, 15, 30, 60, 90 (gray lines), and 120 min (blue line) prior to the addition of excess dithionite (dashed red line). B) Spectra of an MtrC (26 μm)/eosin Y (14 μm) solution as prepared (black line) and after illumination for 5, 15, 30, 60 (gray lines), and 90 min (blue line) prior to the addition of excess dithionite (dashed red line). Arrows indicate the direction of spectral change during illumination (*λ*>390 nm, power ≈400 W m^−2^). Anaerobic samples in 50 mm TEOA, 50 mm HEPES, 2 mm CaCl_2_, 10 mm KCl, pH 7 at 20 °C. Path length: 1 mm.

Anaerobic solutions of 32 μm MtrC displayed spectral features that are typical of the oxidized protein and unchanged by the addition of 10 μm FMN (e.g., Figure 3 A, black line). Illumination of this sample (*λ*>390 nm, power ≈400 W m^−2^; see the Experimental Section) resulted in the appearance of peaks with maxima at 523 and 552 nm indicative of ferrous heme (Figure 3 A, gray lines). The magnitudes of these peaks, which represent the extent of MtrC reduction, increased to a maximum during 90 min illumination and were unchanged by 30 min further illumination (Figure 3 A, blue line). A similar experiment in the absence of FMN provided no evidence for heme reduction (Figure S2 A). Thus, FMN was revealed to be an effective photosensitizer for the visible‐light‐driven reduction of heme groups within MtrC.

The extent of heme photoreduction was quantified by addition of excess sodium dithionite (e.g., Figure 3 A, dashed red line). Dithionite (S_2_O_4_^2−^) has a reduction potential of about −500 mV (all potentials quoted vs. SHE) under the conditions of our study,^18^ and when present in excess it reduces all ten MtrC heme groups.^6^, ^15^, ^19^ As a consequence, the electronic absorbance of fully reduced MtrC at 552 nm was compared with that of the MtrC generated by FMN‐dependent photoreduction, and for the example shown in Figure 3 A photoreduction was found to proceed to 56 %. Similar results were obtained in repeat experiments and for ratios of FMN to MtrC that ranged from approximately 1:0.005 to 1:1.5 with 10 or 100 μm MtrC (Table 1). Thus, the extent of MtrC photoreduction was independent of whether the concentration of FMN exceeded that of MtrC or was catalytic (sub‐stoichiometric) with respect to the protein.

**Table 1:** Photoreduction of MR‐1 cytochromes by the indicated photosensitizers.

	Extent of heme reduction [%]		
Photosensitizer	MtrC	OmcA	MtrCAB
RF^[a]^	60±6^[b]^ (*n*=2)	63±5^[b]^ (*n*=5)	62±6^[c]^ (*n*=2)
FMN^[a]^	61±5^[b]^ (*n*=4)	59±5^[c]^ (*n*=2)	58±5^[c]^ (*n*=2)
FAD^[a]^	62±5^[b]^ (*n*=2)	66±8^[b]^ (*n*=3)	61±5^[c]^ (*n*=2)
fluorescein^[a]^	100±2^[b]^ (*n*=3)	100±2^[b]^ (*n*=4)	100±2^[c]^ (*n*=4)
proflavine^[a]^	100±2^[b]^ (*n*=3)	100±2^[b]^ (*n*=4)	100±2^[c]^ (*n*=2)
eosin Y^[a]^	100±2^[b]^ (*n*=4)	100±2^[b]^ (*n*=4)	100±2^[c]^ (*n*=4)
[Ru(bpy)_3_]^2+[a]^	≈2^[d]^ (*n*=2)	14±6^[b]^ (*n*=2)	≈2^[e]^ (*n*=2)
RuP^[a]^	≈2^[d]^ (*n*=2)	≈1^[d]^ (*n*=2)	≈3^[e]^ (*n*=2)
RuP**⋅**TiO_2_ **⋅**MtrC^[f]^	76±10 (*n*=3)	84±4 (*n*=3)	78±13 (*n*=3)
RuP**⋅**TiO_2_ **⋅**OmcA^[f]^	82±12 (*n*=2)	75±15 (*n*=2)	49±25 (*n*=2)
none	n.d.^[g]^ (*n*=2)	n.d.^[g]^ (*n*=2)	3±2 (*n*=2)

[a] Experiments performed in anaerobic 50 mm TEOA, 50 mm HEPES, 2 mm CaCl_2_, 10 mm KCl, pH 7 at 20 °C and with Triton X‐100 (0.06 %, *v*/*v*) included for MtrCAB. Extent of reduction during 90 min illumination (*λ*>390 nm, power 400 W m^−2^) is the average of *n* experiments where the error is the difference between the average and the maximum and minimum values. Heme reduction calculated from absorbance at 552 nm except for eosin Y when present in excess because the photosensitizer absorbance meant that the heme oxidation state was more clearly assessed at 420 nm. [b] Photosensitizer/protein ratios from 1:0.005 to 1:1.5 with 10 μm and 100 μm photosensitizer. [c] Photosensitizer/protein ratios from 0.5:0.005 to 0.5:1.5 with 10 μm and 100 μm photosensitizer. With 20 and 10 heme groups per MtrCAB and MtrC(OmcA), respectively, this ensured comparable optical densities for the experiments with each protein. [d] 100 μm photosensitizer, 0.5 μm protein. [e] 100 μm photosensitizer, 0.25 μm MtrCAB. [f] Experiments performed in anaerobic 150 mm, pH 6 at 20 °C with Triton X‐100 (0.2 %, *v*/*v*) included with MtrCAB. Particles (0.037 mg mL^−1^) with 0.65 μm MtrC, 0.61 μm OmcA, or 0.31 μm MtrCAB. Extent of reduction during 60 min illumination (*λ*>390 nm, power 400 W m^−2^) is the average of *n* experiments for which the error is the difference between the average and the maximum and minimum values. Heme reduction calculated from absorbance at 552 nm. [g] n.d.: none detected.

Results very similar to those described above were obtained in parallel experiments with MtrC replaced by OmcA, and also when FMN was replaced by RF or FAD with either protein (Table 1). However, 100 % photoreduction of MtrC and OmcA was triggered by 90 min illumination of samples that contained eosin Y, proflavine, or fluorescein (e.g., Figure 3 B and Table 1). As in the cases of FMN, RF, and FAD, during the 90 min a steady‐state level of cytochrome photoreduction was reached; this was independent of whether eosin Y, proflavine, or fluorescein were present either in catalytic quantities or in excess. In contrast, less than 15 % photoreduction of the cytochromes was observed during 90 min illumination with either [Ru(bpy)_3_]^2+^ or RuP present even at ≈200‐fold excess over protein, and the photoreduction failed to reach steady‐state conditions (Table 1).

**Figure 4:**
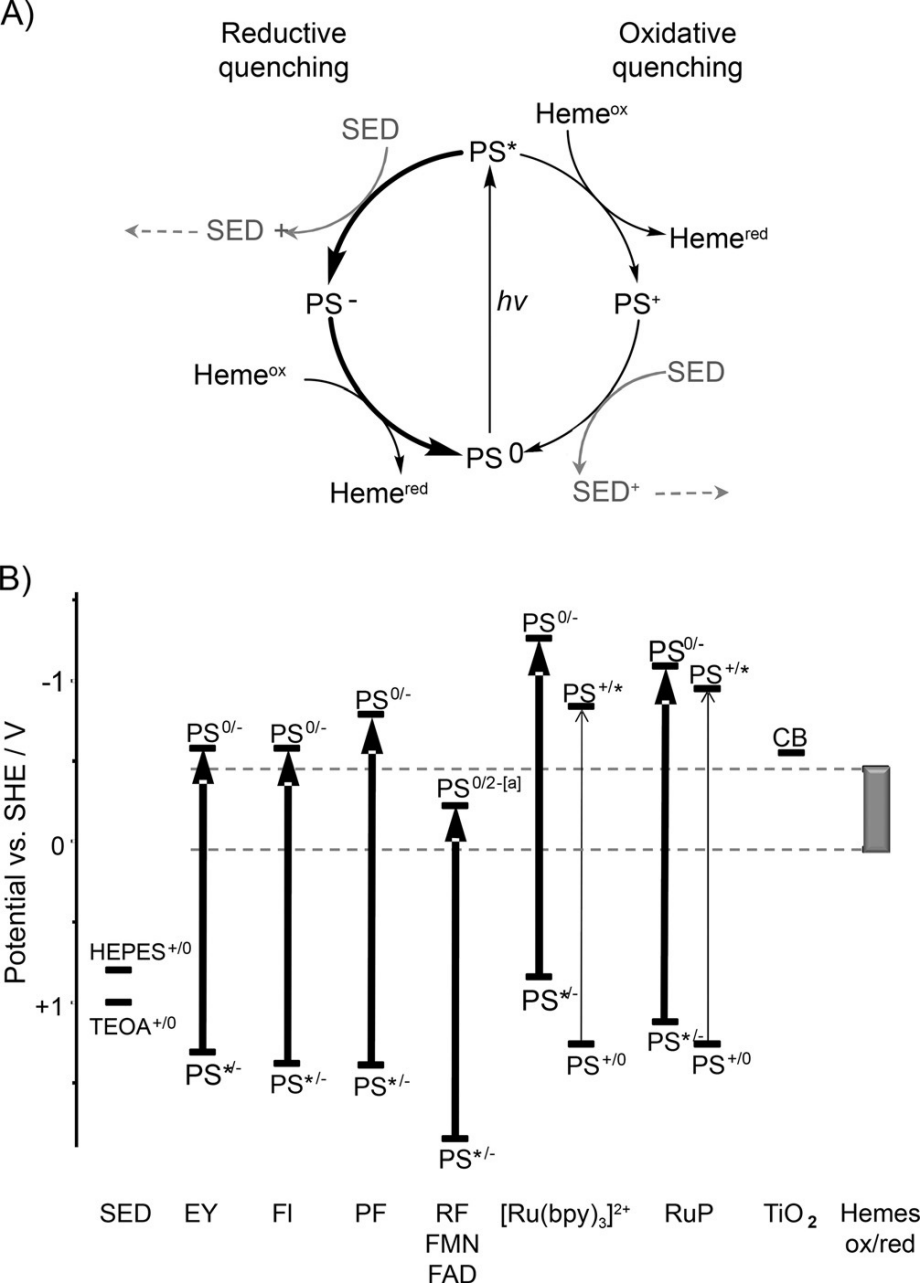
Overview of photochemistry relevant to the photosensitizers employed in this study. A) Pathways of oxidative and reductive quenching after light absorption by a photosensitizer (PS). The ground (PS^0^), photoexcited (PS*), one‐electron‐reduced (PS^−^), and one‐electron‐oxidized states (PS^+^) are indicated. SED is the sacrificial electron donor. For simplicity only those steps that contribute directly to photoreduction are shown. B) (Photo)reduction potentials relevant to photoreduction of the MR‐1 cytochromes by eosin Y (EY), fluorescein (Fl), proflavine (PF), RF, FMN, FAD, [Ru(bpy)_3_]^2+^, RuP at pH 7, and the conduction band (CB) of TiO_2_ nanoparticles at pH 6 (see text and Table S1 for details). Thick (thin) arrows relate to processes of reductive (oxidative) quenching. Potentials relevant to oxidation of HEPES and TEOA are indicated, together with the window spanned by the reduction potentials of the heme groups (Heme^ox/red^) for each of MtrC, OmcA, and MtrCAB. [a] PS^0/2−^ for RF, FMN, and FAD denotes the quinone/hydroquinone couple.

To gain insight into the extent of photoreduction observed after 90 min illumination with each photosensitizer (PS) it was of interest to identify the corresponding photocatalytic cycle(s). Photoreduction of a protein can in principle be associated with oxidative and reductive quenching^20^ of a photoexcited state (PS*, Figure 4 A). The protein substrate would be directly reduced when PS* is oxidatively quenched. The product (PS^+^) would then be regenerated to the ground state (PS^0^) through oxidation of a sacrificial electron donor (SED). During reductive quenching, PS* would first oxidize the SED and form PS^−^. This would then reduce the protein to recover the ground state (PS^0^) and complete the photocatalytic cycle (Figure 4 A). The feasibility of these pathways is determined by the (photo)reduction potentials of the photosensitizer relative to those of the SED and protein (e.g., Figure 4 B and Table S1). In practice the rates of these reactions and those of competing processes, productive or not with respect to reduction of the protein, will define the extent of photoreduction under any given conditions.^20^ Oxidative and reductive quenching of organic photosensitizers typically produce radical species, and as a consequence their photochemical behavior often extends beyond that illustrated in Figure 4 A.

**Figure 5:**
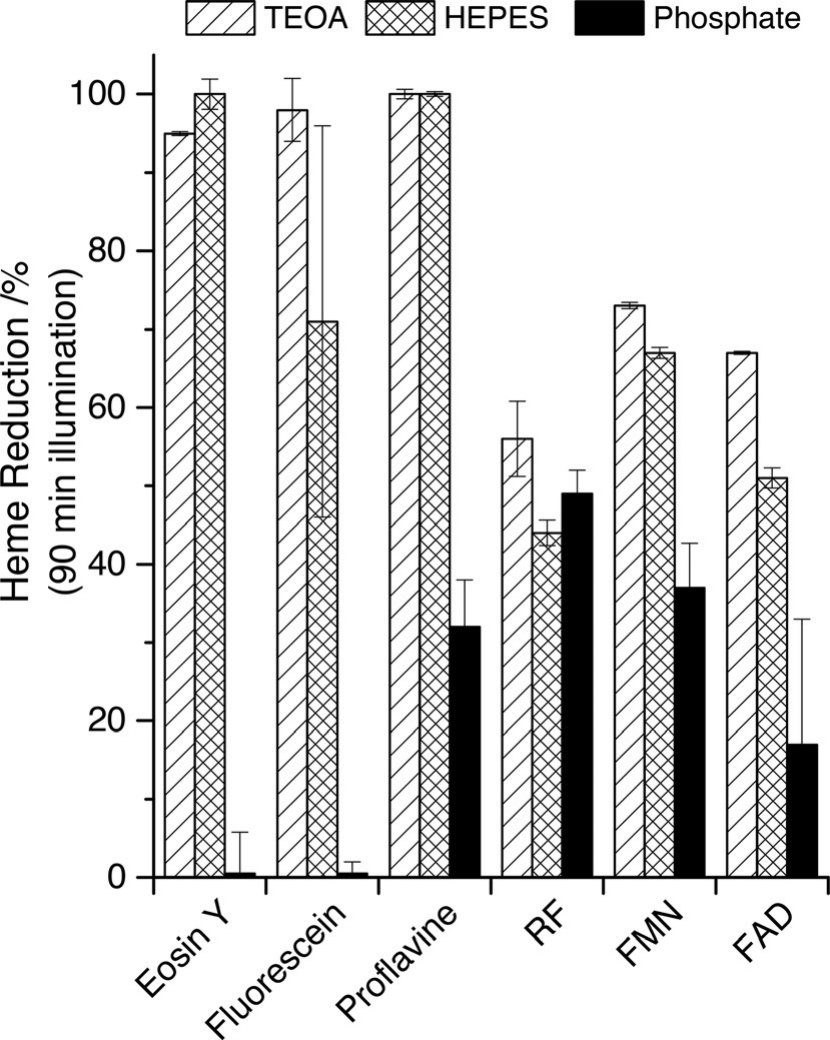
Impact of SED on the extent of OmcA photoreduction triggered by 90 min illumination. Extent of heme reduction (%) after 90 min illumination (*λ*>390 nm, power ≈400 W m^−2^) of 0.5 μm OmcA at pH 7, 20 °C with 10 μm of each indicated photosensitizer in anaerobic 50 mm phosphate (inactive as SED) or with 50 mm TEOA, or 50 mm HEPES, as indicated. Heme reduction was quantified by change in electronic absorbance at 552 nm except in the case of eosin Y, in which spectral overlap of contributions from the photosensitizer and protein at this wavelength necessitated an equivalent analysis for the Soret peak at 420 nm (see text for details). Extents of reduction are the averages of two experiments and the errors are the differences between the average and the maximum and minimum values.

In order to assess the contributions made by TEOA and HEPES as SEDs during photoreduction of the extracellular cytochromes, OmcA (0.5 μm) was illuminated for 90 min with a 20‐fold excess of a given photosensitizer in a 50 mm phosphate solution and, in separate experiments, with TEOA or HEPES. Phosphate is inactive as a SED, and we chose to restrict these studies to OmcA, because MtrC and OmcA have very similar structures, thermodynamic properties,8b, 19 and, as we have shown here, photochemical behavior (Table 1). Eosin Y with TEOA (or HEPES) supported 100 % photoreduction of OmcA (Figure 5). However, no photoreduction was detected in the absence of the tertiary amines, which suggests that OmcA reduction is coupled to reductive quenching of the eosin Y photoexcited state by the SED. Under conditions comparable to those used here the corresponding PS^0/−^ couple has a reduction potential (*E*_m_) of ≈−580 mV.^21^

The *E*_m_ values for the OmcA heme groups span from approximately +50 to −450 mV at the neutral pH of these experiments and they are relatively evenly distributed across this potential window.^19^ The same is true of the corresponding values for the MtrC heme groups.^6^, ^13^ Thus, the observed 100 % photoreduction of OmcA, and of MtrC, by eosin Y supported by TEOA and/or HEPES is consistent with the relevant (photo)reduction potentials (Figure 4 B). Similarly, the pattern of fluorescein‐dependent OmcA photoreduction indicates that reductive, but not oxidative, quenching operates (Figure 5). Reductive quenching of fluorescein under conditions comparable to those in our studies produces a reductant^21^ with sufficient driving force to reduce OmcA and MtrC completely (Figure 4 B). That this occurs with TEOA but not with HEPES indicates additional complexity in the latter system.

Previous studies have revealed complex photochemistry of proflavine under conditions comparable to those employed here.20a, 20b In anaerobic aqueous solutions reductive quenching predominates when a SED is present. However, in the absence of a SED the corresponding PS* decays by triplet–triplet annihilation and photoionization to produce a solvated electron. The proflavine‐dependent photoreduction of OmcA proceeded more effectively when TEOA or HEPES were present (Figure 5) and presumably occurred through reductive quenching. The PS^0/−^ couple relevant to this pathway has a sufficiently negative reduction potential20c to account for the complete photoreduction of OmcA and MtrC observed when the SEDs are present (Figure 4 B).

For RF, FMN, and FAD it is generally accepted that the corresponding PS* is reductively quenched to yield a semiquinone that undergoes rapid disproportionation to generate a hydroquinone, this being the active photoreductant.20d, 20e Both inter‐ and intramolecular electron transfer reactions lead to reductive quenching20d, 20e and this is consistent with the cytochrome photoreduction that was observed in the absence of TEOA or HEPES as SED in our experiments (Figure 5). The hydroquinone forms of RF, FMN, and FAD have *E*_m_ values in the middle of the range spanned by the heme groups of OmcA and MtrC (Figure 4 B). Thus, in the presence of TEOA and HEPES the steady‐state levels of cytochrome photoreduction (≈60 %, Table 1) produced by these photosensitizers in comparison with the complete reduction produced by eosin Y, fluorescein, or proflavine were consistent with consideration of the available driving forces. However, this was not the case for [Ru(bpy)_3_]^2+^ and RuP, for which significantly less photoreduction was observed. Oxidative and reductive quenching of the corresponding photoexcited states would generate stronger reductants20f, 22 than the photocatalytic cycles operative with any of the organic photosensitizers studied here (Figure 4 B). Because negligible photoreduction of the extracellular cytochromes was induced by the Ru^II^ dyes their effectiveness as photosensitizers relative to the organic dyes must be compromised, by, for example, slow net electron exchange with the cytochromes and SED and/or nonproductive side reactions. Indeed, greater levels of OmcA and MtrC photoreduction were observed over 5 h illumination (e.g., Figure S2 B and C).

Two additional series of experiments were performed to quantify the behavior of MtrC under conditions that might be relevant to those on the surface of MR‐1. The first series of experiments quantified photoreduction of the heme groups in detergent‐solubilized MtrCAB complex purified from the MR‐1 outer membrane (Figure 1 A). MtrCAB is a stable 1:1:1 complex of the MtrC, MtrA, and MtrB proteins that contains 20 heme groups with *E*_m_ values spanning a potential window similar to those of MtrC and OmcA.^6^, ^19^ Suspensions of MtrCAB illuminated with each of the photosensitizers discussed above, TEOA, and HEPES produced behavior similar to that displayed by MtrC and OmcA alone (Table 1). As a consequence, the response of MtrCAB to these photosensitizers is most likely determined by the same factors as for the extracellular cytochromes alone. Finally, in view of recent reports that MtrC might exist as a flavocytochrome on the surface of MR‐1,^9^ it was of interest to characterize the response of such a protein to illumination with visible light. Flavocytochrome, composed of FMN tightly bound to MtrC, was prepared as described previously, by anaerobic incubation of MtrC with glutathione and FMN, followed by gel filtration to separate the flavocytochrome from free FMN.8b Electronic absorbance spectroscopy established that the heme groups and FMN remained oxidized throughout these processes, which were performed in the dark, and also after 90 min illumination (Figure S3). Previous studies have established that the fluorescence of FMN is partially quenched on binding to the glutathione‐reduced MtrC and that this fluorescence is recovered when the FMN is released by oxidation of the flavocytochrome.8b This quenching most likely occurs through FRET due to the proximity of the bound FMN to heme. We suggest that bound FMN does not support photoreduction of the flavocytochrome because the corresponding excited state is quenched more rapidly by energy transfer than by electron transfer. It is proposed that the observed photoreduction of MtrC by solutions of FMN, and presumably also RF or FAD (Table 1), occurs through electron transfer at a site other than that occupied by the flavin in the flavocytochrome.

### Photoreduction of MtrC and OmcA adsorbed on dyesensitized TiO_2_ nanoparticles

A previous study reported negligible intermolecular transfer of photoenergized electrons from RuP to a molecular cobalt catalyst in a pH‐neutral aqueous solution in the presence of a SED.^23^ However, co‐adsorption of this dye and catalyst on TiO_2_ nanoparticles promoted efficient light‐driven H_2_ evolution. Charge separation from photoexcited RuP was enhanced through rapid oxidative quenching by TiO_2_, and photoenergized electrons in the conduction band were readily transferred to the catalyst. The TiO_2_ conduction band has a reductive potential of approximately −620 mV at pH 6^24^ such that complete photoreduction of MtrC and OmcA by RuP‐coated TiO_2_ particles (RuP**⋅**TiO_2_), is thermodynamically feasible (Figure 4 B). As a consequence it was attractive to establish whether MtrC or OmcA would adsorb on RuP**⋅**TiO_2_ in an electroactive configuration (Figure 2 B).

**Figure 6:**
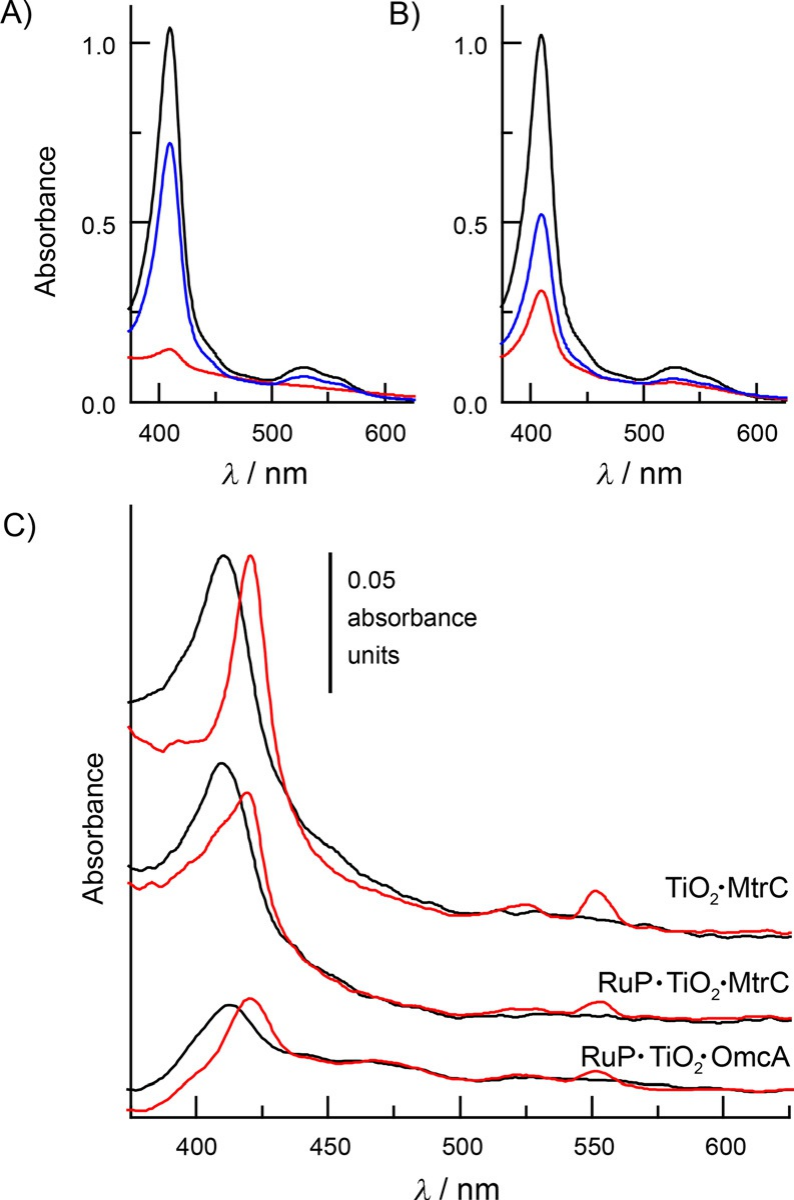
Adsorption and photoreduction of MtrC and OmcA on (RuP**⋅**)TiO_2_ particles. Electronic absorbance of A) MtrC and B) OmcA solutions (1 μm, 200 μL) before (black) 30 min incubation with TiO_2_ particles (0.5 mg mL^−1^) in 150 mm MES (pH 6) followed by centrifugation to pellet the particles and retrieval of the supernatant (red). The protein‐coated particles were resuspended in 200 μL of 25 mm phosphate, 150 mm MES (pH 6) and incubated for 30 min to release the bound protein. The particles were pelleted by centrifugation, and spectroscopy of the supernatant (blue) quantified the released protein. C) Electronic absorbance recorded with an integrating‐sphere spectrophotometer for suspensions (0.055 mg mL^−1^) of (RuP**⋅**)TiO_2_ **⋅**MtrC (OmcA) particles as indicated. Spectra are presented after subtraction of the response from an equivalent solution of unmodified particles and offset on the y‐axis for clarity; TiO_2_ **⋅**MtrC particles before (black) and after (red) addition of excess sodium dithionite (top), RuP**⋅**TiO_2_ **⋅**MtrC particles before (black) and after (red) 20 min illumination (middle), RuP**⋅**TiO_2_ **⋅**OmcA particles before (black) and after (red) 10 min illumination (bottom). Experiments performed in anaerobic 150 mm MES (pH 6), 20 °C. Illumination *λ*>390 nm, power≈ 400 W m^−2^.

RuP forms a stable linkage with TiO_2_ at slightly acidic pH by virtue of its phosphonic acid groups.^25^ The ability of OmcA or MtrC to adsorb on P25 TiO_2_ nanoparticles under these conditions was assessed when each protein (1 μm) in 200 μL of 150 mm SED MES at pH 6 was incubated with 0.5 mg mL^−1^ particles. After 30 min incubation with occasional inversion the samples were centrifuged to pellet the particles and any adsorbed cytochrome. The white particles were found to have taken on a pink‐red color after incubation with the proteins (Figure S4). The amount of cytochrome that remained in the supernatant was quantified by electronic absorbance spectroscopy and found to be significantly less than that in the protein solution prior to incubation with the particles (Figure 6 A, B). Both observations were consistent with protein adsorption on the TiO_2_ particles.

The integrity of the adsorbed proteins was confirmed by electronic absorbance of the protein‐coated (TiO_2_ **⋅**MtrC(OmcA)) particles measured with an integrating‐sphere spectrophotometer (e.g., Figure 6 C, top). Use of the integrating sphere mitigated against loss of incident light due to scattering by the particles and facilitated the resolution of spectral features from the adsorbed proteins. The corresponding spectra displayed features typical of the oxidized proteins: namely, a strong absorbance in the Soret region with a maximum at 420 nm and a broader, less intense feature in the αβ region between 500 and 600 nm. These features disappeared on addition of excess dithionite, when peaks with maxima at 420 and 552 nm characteristic of the fully reduced proteins were revealed (e.g., Figure 6 C top). Thus, the adsorbed proteins retained the spectral properties and redox activities of their soluble counterparts.

To assess the longevity of protein adsorption, TiO_2_ **⋅**MtrC(OmcA) particles were suspended in a fresh 200 μL solution of 150 mm MES (pH 6) and incubated with occasional inversion for 20 h at 4 °C. The particles were then pelleted by centrifugation, and electronic absorbance spectroscopy of the supernatant provided no evidence for protein desorption. In contrast, protein was clearly present in supernatant recovered from centrifugation of TiO_2_ **⋅**MtrC(OmcA) particles incubated for 30 min at 4 °C in 200 μL of 25 mm phosphate, 150 mm MES, pH 6. Recovered particles were white. Electronic absorbance spectra of the desorbed proteins provided no evidence for perturbation of protein structure by adsorption (Figure 6 A, B, blue). It was concluded that adsorption of MtrC and OmcA in their native states was tight and essentially irreversible in 150 mm MES at pH 6 but that the binding was reversed under conditions of competitive phosphate binding.

The maximum extents of protein adsorption were estimated from the differences in electronic absorbance of protein solutions before and after incubation with TiO_2_ particles, and from the electronic absorbance of the protein released from the particles on exposure to the phosphate‐containing buffer (Figure 6 A, B). Both methods quantified the amount of MtrC and OmcA that had been adsorbed as approximately 1.8 and 1.4 nmol, respectively, per mg of TiO_2_ nanoparticles. Taking the surface area of the particles as 50 m^2^ g^−1^ and the dimensions of MtrC and OmcA to be approximately 4×6×8 nm and 5×6×10 nm, respectively,^8^ indicated that both proteins adsorbed at close to monolayer coverage.

The stable adsorption of MtrC(OmcA) on TiO_2_ having been established, the possibility of visible‐light‐driven reduction of the adsorbed cytochromes by RuP‐sensitization of the TiO_2_ (Figure 2 B) was assessed. In the desired configuration, adsorbed RuP should pass photoexcited electrons to adsorbed protein through the conduction band of the TiO_2_. RuP was consequently first adsorbed on the P25 TiO_2_ particles to 20 % of its maximal coverage as described in the Experimental Section. The RuP**⋅**TiO_2_ particles were then exposed to sufficient MtrC(OmcA) to saturate the sites that were available for protein adsorption. Washed RuP**⋅**TiO_2_ **⋅**MtrC(OmcA) particles were then resuspended in anaerobic 150 mm MES (pH 6) and illuminated (*λ*>390 nm, 400 mW m^−2^). After this illumination the electronic absorbance spectra showed a clear red shift of the Soret maximum and peaks with maxima at 420 and 552 nm (e.g., Figure 6 C, middle, bottom). These spectral changes revealed visible‐light‐driven reduction of MtrC(OmcA) adsorbed on the TiO_2_ particles sensitized with RuP.

### Photocatalytic reduction of solutions of MtrC, OmcA and MtrCAB by RuP⋅TiO_2_⋅MtrC(OmcA) particles

**Figure 7:**
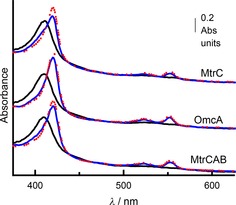
Photoreduction of MtrC, OmcA, and MtrCAB solutions by RuP**⋅** TiO_2_ **⋅**MtrC particles. Electronic absorbance of RuP**⋅**TiO_2_ **⋅**MtrC suspensions (0.037 mg mL^−1^) with 0.65 μm MtrC (top), 0.61 μm OmcA (middle), and 0.31 μm MtrCAB (bottom) before (black) and after (blue) 30 min illumination (*λ*>390 nm, power ≈400 W m^−2^), followed by the addition of excess sodium dithionite (red). Experiments performed at 20 °C in anaerobic 150 mm MES (pH 6) with Triton X‐100 (0.2 %, *v*/*v*) included for MtrCAB. Spectra offset on the *y*‐axis for clarity.

A motivation for this work was to inform strategies that might allow extracellular photosensitizers to generate photoenergized electrons that can enter MR‐1 with the aid of extracellular and outer‐membrane‐associated cytochromes (Figure 1 A), in order to drive reductive catalysis by intracellular enzymes. For the RuP**⋅**TiO_2_ **⋅**MtrC(OmcA) particles this requires that the adsorbed proteins be able to pass electrons to redox partner proteins in a photocatalytic process (Figure 2 C). Given that the coverage of the TiO_2_ by MtrC(OmcA) approached that predicted for a monolayer, and that no desorption was detected over 20 h in 150 mm MES (pH 6) (see above), we reasoned that the protein‐coated particles offered very little opportunity for direct electron exchange to occur from the surface of the RuP**⋅**TiO_2_ particles to proteins in solution. As a consequence, stirred, anaerobic suspensions of RuP**⋅**TiO_2_ **⋅**MtrC particles (0.037 mg mL^−1^) were added to solutions of MtrC, OmcA, or MtrCAB (≈6.2 μm heme) and illuminated to seek evidence for electron transfer through the adsorbed proteins to protein molecules in solution. Electronic absorbance spectroscopy revealed 75–85 % heme reduction in each solution over 60 min illumination (Figure 7, Table 1, and Figure S5). Particles recovered by centrifugation at the end of the experiment had a red color, and no reduction was observed when the experiment was repeated without illumination. It was concluded that the RuP**⋅**TiO_2_ **⋅**MtrC particles performed photocatalytic reduction of the cytochrome solutions (ca. one RuP particle per 21 heme groups). Parallel experiments established that RuP**⋅**TiO_2_ **⋅**OmcA particles performed photocatalytic reduction of MtrC, OmcA, and MtrCAB (Table 1 and Figure S7).

In principle, electron transfer from RuP to MtrC(OmcA) on the particles could occur by two pathways:20f through the conduction band of the TiO_2_ or by direct RuP‐to‐protein electron transfer. Results from a final series of experiments were consistent with electron transfer through the TiO_2_ conduction band. No evidence for photoreduction was observed when solutions of RuP and protein were illuminated in the absence of TiO_2_ particles [RuP in 175‐fold excess of MtrC, RuP with MtrC (OmcA or MtrCAB) at concentrations equivalent to those in the particle‐containing suspensions, Figure S6]. Similarly, no photoreduction was detected when 25 mm phosphate was included in suspensions that contained RuP, TiO_2_, and MtrC because phosphate at these concentrations adsorbed on the TiO_2_ in preference to RuP and MtrC (see above). Direct TiO_2_‐to‐protein electron transfer was demonstrated when TiO_2_ **⋅**MtrC particles were seen to catalyze photoreduction of MtrC solutions when illuminated by UV light that excited electrons across the TiO_2_ band gap (Figure S8). These particles failed to catalyze photoreduction when illuminated by visible light (Figure S8), due to the absence of RuP that can be photoexcited by d‐to‐π* metal‐to‐ligand charge‐transfer transition.^23^, ^24^, ^25^, ^26^ Taken together these results are consistent with facile visible‐light‐driven reduction of MtrC(OmcA) after charge injection into the conduction band of TiO_2_ initiated by photoexcitation of the RuP. In agreement with this conclusion, use of the integrating‐sphere spectrophotometer showed no evidence for photoreduction of the MtrC adsorbed on TiO_2_ particles in the absence of RuP during 60 min illumination with *λ*>390 nm (Figure S9). This experiment also revealed a time‐dependent decrease in the apparent absorbance through the Soret region (<450 nm) that is most likely to arise from changes in light scattering by the particles. This behavior, rather than damage to the protein, can account for the spectral differences displayed by suspensions of (RuP**⋅**)TiO_2_ **⋅**MtrC particles in relation to solutions of MtrC, Figures 6 C and 7.

### Prospects for light‐driven microbial synthesis

Enzymes are excellent catalysts for processes very relevant to developing the production of solar fuels and chemicals.^4^, ^26^, ^27^ However, time‐consuming and costly purification procedures, together with limited stability of the isolated proteins, often present bottlenecks to their effective utilization. By performing catalysis inside bacteria the need for enzyme purification is removed and catalyst self‐repair and regeneration might be possible.^4^, ^27^ Furthermore, there are opportunities to improve on the efficiencies of natural photosynthetic processes^28^ by employing extracellular photosensitizers designed, for example, to absorb light across the solar spectrum and to deliver photoenergized electrons to intracellular enzymes through EET pathways. A framework for informed development of light‐driven microbial synthesis in MR‐1 and heterologous hosts^14^ is provided by the photoreduction of the MR‐1 extracellular and outer‐membrane‐spanning cytochromes demonstrated here.

MtrCAB, MtrC, and OmcA are redox‐active over similar windows of electrochemical potential, and their complete photoreduction is triggered by eosin Y, fluorescein, and proflavine when combined with an appropriate SED. Comparable reduction of MtrCAB is achieved by dithionite,^6^ and when MtrCAB spans the bilayer of a lipid vesicle it couples oxidation of external dithionite to reduction of internalized methyl viologen.^10^ As a consequence it is thermodynamically feasible that the eosin‐Y‐, proflavine‐, and fluorescein‐dependent photoreduction of MR‐1 extracellular cytochromes would support strongly endoergic intracellular reactions, because reduced methyl viologen drives the reduction of CO_2_ and water in well‐established biochemical assays. The same thermodynamic predictions are made for the RuP**⋅**TiO_2_‐dependent cytochrome reductions because these particles catalyze light‐driven proton and CO_2_ reduction by molecular catalysts and metalloenzymes.^26^ Facile intracellular electrosynthesis of succinate by MR‐1^29^ is driven by electron transfer from MtrCAB to a periplasmic fumarate reductase (*E*_fumarate/succinate_≈20 mV at pH 7). If the effective light‐driven microbial synthesis of additional products is to be achieved similarly, facile electron transfer to appropriate enzymes through natural or engineered^14^ pathways will be required.

Clearly, the effective translation of information gained in these studies into solar microbial synthesis by MR‐1, or by bacteria engineered to contain the MR‐1 outer‐membrane‐associated cytochromes, requires a consideration of many factors, not least the viability of the bacteria in the presence of photocatalytic concentrations of the photosensitizers. In this regard it is significant that FMN and RF are secreted by MR‐1 and enhance EET.^11^ It is also of note that MR‐1 retains the ability to grow and to secrete RF in the presence of P25 TiO_2_ particles.^30^ As a consequence, experiments that explore the possibilities of employing the photosensitizers described here for visible‐light‐driven synthesis by MR‐1 are ongoing in our laboratories. Given that the outer‐membrane cytochromes of MR‐1 evolved to deliver electrons to extracellular Fe^III^‐containing mineral particles, an alternative strategy for their photoreduction could employ nanocrystalline, semiconductive Fe^III^ oxide particles^31^ as photosensitizers. It will be of interest to establish whether such particles with appropriate conduction band energies and optical properties can transfer electrons to MR‐1 outer‐membrane cytochromes.

## Conclusion

The extracellular and outer‐membrane‐spanning cytochromes MtrC, OmcA, and MtrCAB of MR‐1 can be reduced in photocatalytic processes by water‐compatible photosensitizers. Complete reduction of the cytochromes was achieved with abiotic eosin Y, proflavine, and fluorescein with TEOA as SED, whereas cytochrome photoreduction plateaued at 60 % for biotic RF, FMN, and FAD with TEOA as SED. Under comparable conditions, solutions of abiotic Ru[bpy]_3_^2+^ and RuP sustained very slow cytochrome reduction. This kinetic limitation was overcome by adsorption of MtrC(OmcA) on RuP‐sensitized P25 TiO_2_ nanoparticles that were able to perform photocatalytic reduction of solutions of MtrC, OmcA, and MtrCAB. These findings provide a framework for informed development of strategies to use the outer‐membrane‐associated cytochromes of MR‐1 for solar microbial synthesis in natural and engineered bacteria.

## Experimental Section

**Reagents**: Flavin mononucleotide (FMN, sodium salt), flavin adenine dinucleotide (FAD, disodium salt hydrate), riboflavin (RF), eosin Y (2′,4′,5′,7′‐tetrabromofluorescein), proflavine (3,6‐diaminoacridine, hemisulfate salt), and [Ru(bpy)_3_]Cl_2_ (bpy=2,2′‐bipyridine), all from Sigma–Aldrich, were used without further purification, as was fluorescein (Alfa Aesar). [Ru(bpy)_2_{4,4′‐(PO_3_H_2_)_2_bpy}]Br_2_ (RuP) was prepared as previously described.^32^ Stock solutions of photosensitizers and triethanolamine (TEOA) were prepared by dissolving the appropriate mass in HEPES (pH 7, 50 mm)/CaCl_2_ (2 mm)/KCl (10 mm) or in phosphate (pH 7, 50 mm). Photosensitizer solutions were stored in the dark, and their integrities were confirmed by electronic absorbance spectroscopy prior to each use. P25 TiO_2_ Aeroxide particles [surface area (50±15) m^2^ g^−1^, 21 nm average diameter] were a gift from Evonik Industries. All other reagents were Analar quality or equivalent, and water was of resistivity >18 MΩ cm.

**Proteins**: Soluble forms of MtrC and OmcA from *S. oneidensis* MR‐1 were purified as previously described.^8^ Then, because both proteins were expected to contain a 45‐residue C‐terminal extension that included a His tag, the samples were passed through a Ni‐NTA column in HEPES (pH 7.6, 20 mm)/NaCl (100 mm). Most protein in samples of MtrC or OmcA failed to bind to the column, and that material, after buffer exchange, was used for the experiments reported here. LC‐MS revealed molecular weights for the proteins that were consistent with the absence of the His tag, most likely due to proteolytic cleavage [MtrC: observed mass 75 047 Da (predicted 75 047 Da with no C‐terminal extension); OmcA: observed mass 82 847 Da (predicted 86 319 and 82 279 Da, respectively, with and without the C‐terminal extension)]. The FMN flavocytochrome forms of MtrC8b and MtrCAB,^33^ each as a suspension in Triton X‐100 (2 %, *v*/*v*), were prepared as previously described. Purified proteins were stored at −80 °C, and their concentrations were defined by use of electronic absorbance spectroscopy of the oxidized (air‐equilibrated) proteins at 410 nm with extinction coefficients [mm ^−1^ cm^−1^] of 1000, 1000, and 2000 for MtrC, OmcA, and MtrCAB respectively.

**Experiments with solutions of soluble photosensitizers**: Anaerobic solutions containing the desired protein with eosin Y, proflavine, fluorescein, RF, FMN, or FAD were prepared to explore the possibility of protein photoreduction by soluble photosensitizers (Figure 2 A). Experiments were performed in a N_2_‐filled chamber (Belle Technology, clear acrylic chamber with atmospheric O_2_<10 ppm), and samples were illuminated from outside the chamber with a KL5125 Cold 150 W light source with high‐quality UV filter quartz glass (Krüss) fitted with a 150 W (15 V) halogen lamp (Osram). As a consequence the walls of the N_2_ chamber served as an additional filter for light of wavelengths <400 nm. Light intensity at the sample was calibrated by use of a SOLAR‐100 Amprobe solar power meter (sensor wavelength 400–1100 nm). Stirred solutions of soluble photosensitizers and cytochromes at 5 μm heme concentration were illuminated in 1 cm light path cuvettes (Starna Scientific, special optical glass with >75 % transmission above 320 nm). The high optical densities and quantities of proteins required for experiments with >100 μm total heme precluded easy access to higher volume approaches. Instead these experiments were performed in unstirred solutions with 1 mm path length cuvettes (Starna Scientific, special optical glass). Experiments that explored the mechanism(s) of OmcA photoreduction were performed at pH 7 in phosphate (50 mm) or TEOA (50 mm)/phosphate (50 mm) or HEPES (50 mm)/CaCl_2_ (2 mm)/KCl (10 mm) as indicated. Electronic absorbance spectra were measured with a Biochrom WPA Biowave II diode array spectrophotometer located inside the N_2_‐filled chamber.

**Preparation and characterization of RuP‐sensitized P25 TiO_2_ particles coated with MtrC or OmcA**: A stock dispersion of P25 TiO_2_ nanoparticles [2 mg mL^−1^ in MES (pH 6, 150 mm), stored at 4 °C] was prepared as follows to ensure minimal aggregation in the wash steps. Particles (10 mg) were suspended in MilliQ water (1 mL), sonicated for 1 min, and recovered as a pellet after 5 min centrifugation (9000 *g*, 4 °C). The particles were then subjected to five rounds of resuspension, sonication, and centrifugation as above, in which the resuspensions were into MilliQ water (1 mL, rounds 1 and 2), then MES (pH 6, 150 mm, 1 mL, rounds 3 and 4), and finally MES (pH 6, 150 mm, 5 mL). Particles saturated with adsorbed RuP were prepared essentially as previously described.^34^ In short, the stock dispersion of particles was sonicated for 10 min, and an aliquot was removed and incubated with excess RuP for 30 min on ice with occasional inversion [TiO_2_ (0.1 mg) in RuP (33 μm, 200 μL)/MES (pH 6, 150 mm)]. The RuP‐coated particles were recovered by 5 min centrifugation (9000 *g*, 4 °C), and electronic absorbance of the supernatant quantified non‐adsorbed RuP with use of the extinction coefficient 10.2 mm ^−1^ cm^−1^ at 455 nm (Figure S1). The adsorbed RuP was released by incubation of the particles in phosphate/MES (pH 6, 25 mm and 150 mm, respectively) and quantified through the electronic absorbance of the supernatant after centrifugation to recover the white particles. These procedures confirmed the upper limit of adsorption as approximately 40 nmol RuP per mg TiO_2_ under our conditions. Maximum adsorption of MtrC(OmcA) onto the particles was defined in a similar manner after the desired mass of freshly sonicated particles had been incubated with protein (1 μm, 200 μL)/MES (pH 6, 150 mm) (see the Results). The spectral integrity and redox activity of the adsorbed MtrC(OmcA) was assessed by electronic absorbance spectroscopy with a Hitachi U4100 integrating‐sphere spectrophotometer.

Particles coated with RuP and MtrC(OmcA) (Figure 2 B) were prepared by incubation with sufficient RuP to achieve 20 % maximal coverage, and the resulting particles were exposed to sufficient MtrC(OmcA) to saturate the available binding sites. Freshly sonicated particles (1 mg) were pelleted by centrifugation (9000 *g*, 4 °C, 5 min) and resuspended in RuP (1 mL, 8 μm) in MES (pH 6, 150 mm). After 30 min incubation on ice with occasional inversion, particles with adsorbed RuP were recovered by centrifugation (2200 *g*, 4 °C, 5 min). Electronic absorbance spectroscopy of the supernatant confirmed adsorption of all the previously detectable RuP. Pelleted particles were resuspended in MtrC(OmcA) (2 μm, 1 mL)/MES (pH 6, 150 mm) and incubated for 30 min on ice with occasional inversion. Particles were recovered by centrifugation (2200 *g*, 4 °C, 5 min). Electronic absorbance of the supernatants confirmed that protein adsorption had saturated the available binding sites (typically 90–95 % of the maximum adsorption seen in the absence of RuP). For photoreduction experiments, particles coated with RuP and MtrC(OmcA) were washed by resuspension in MES (pH 6, 150 mm), recovered by centrifugation (2200 *g*, 4 °C, 5 min), and transferred to a N_2_‐filled chamber (as above). Photoreduction of the protein co‐adsorbed with RuP on TiO_2_ particles (Figure 2 B) was assessed with stirred anaerobic suspensions [0.1 mg mL^−1^ in MES (150 mm, pH 6)] in sealed quartz cuvettes (1 cm path length) illuminated as described above. Cuvettes were removed from the N_2_‐filled chamber for measurements of electronic absorbance with a Hitachi U4100 integrating‐sphere spectrophotometer. The ability of particles coated with RuP and MtrC(OmcA) to pass electrons to non‐adsorbed proteins (Figure 2 C) was assessed with the particles (1 mg mL^−1^, 35 μL) resuspended in an anaerobic solution (950 μL) of the desired concentration of MtrC, OmcA, or MtrCAB [detergent in the last case (Triton X‐100, approximately 0.2 %, *v*/*v*)] and MES (pH 6, 150 mm). The stirred samples in sealed glass cuvettes (1 cm path length) were illuminated, and their electronic absorbances were measured inside the N_2_‐filled chamber as described above.

## Supporting information

As a service to our authors and readers, this journal provides supporting information supplied by the authors. Such materials are peer reviewed and may be re‐organized for online delivery, but are not copy‐edited or typeset. Technical support issues arising from supporting information (other than missing files) should be addressed to the authors.

Supplementary
