# Supplementary material for: Photoreduction of Shewanella oneidensis Extracellular Cytochromes by Organic Chromophores and Dye‐Sensitized TiO2
Source: Chembiochem. 2016 Nov 8;17(24):2324–33. doi: 10.1002/cbic.201600339 (PMC5215560; doi:10.1002/cbic.201600339)
Supplement: Supplementary file 1 — Supplementary [file CBIC-17-2324-s001.pdf]

CHEMBIOCHEM

Supporting Information

Photoreduction of *Shewanella oneidensis* Extracellular Cytochromes by Organic Chromophores and Dye-Sensitized TiO_2

Emma V. Ainsworth+,[a] Colin W. J. Lockwood+,[a] Gaye F. White,[b] Ee Taek Hwang,[c] Tsubasa Sakai,[d, e] Manuela A. Gross,[d] David J. Richardson,[b] Thomas A. Clarke,[b] Lars J. C. Jeuken,*[c] Erwin Reisner,*[d] and Julea N. Butt*[a, b]

cbic_201600339_sm_miscellaneous_information.pdf

Table S1. Structural and Photochemical Properties of Photosensitizers (PS) and Sacrificial Electron Donors (SED). PS*, PS^0, PS^+ and PS^- correspond to the states illustrated in Figure 4A main text. Potentials at pH 7 vs SHE. Structures illustrated for the predominant form at pH 7.

	$PS^{0/*}$ (eV)	*E*($PS^{*/-}$) (mV)	*E*($PS^{0/-}$) (mV)	*E*($PS^{0/2-}$) (mV)	*E*($PS^{+/*}$) (mV)	*E*($PS^{+/0}$) (mV)	Ref
Eosin-Y 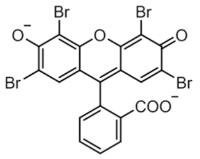	1.89	+1310	-580	n.a.	n.a.	n.a.	1
Fluorescein 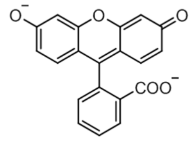	1.96	+1380	-580	n.a.	n.a.	n.a.	1
Proflavine 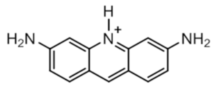	2.17	+1390	-780	n.a.	n.a.	n.a.	2
Flavin 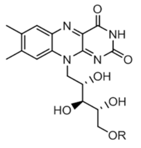 R = H, RF<br>R = PO_3^{2-}, FMN<br>R = $(PO_3^-)(PO_3^-)$-adenosine, FAD	2.07	+1850[a]	n.a.	-220	n.a.	n.a.	3
$[Ru(bpy)_3]^{2+}$ 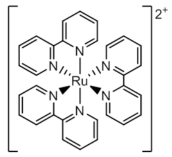	2.10	+840	-1260	n.a.	-840	+1260	4
RuP 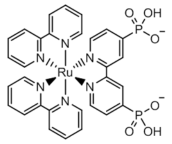	2.21	+1120	-1090	n.a.	-950	+1260	5

[a] For flavin the relevant couple is $PS^{*/2-}$

						$E(SED^{+/0})$ (mV)	Ref
HEPES 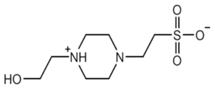	n.a.	n.a.	n.a.	n.a.	n.a.	+800	6
TEOA 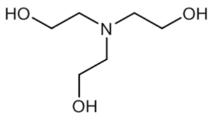						+1000	7

References for Table S1.

1. Chan, M. S.; Bolton, J. R., Structures, reduction potentials and absorption maxima of synthetic dyes of interest in photochemical solar-energy storage studies. *Sol Energy* **1980,** *24* (6), 561-574.
2. (a) Pileni, M. P.; Gratzel, M., Light-induced redox reactions of proflavin in aqueous and micellar solution. *J Phys Chem* **1980,** *84* (19), 2402-2406; (b) Ghosh, T.; Slanina, T.; Konig, B., Visible light photocatalytic reduction of aldehydes by Rh(III)-H: a detailed mechanistic study. *Chem Sci* **2015,** *6* (3), 2027-2034; (c) Kalyanasundaram, K.; Dung, D., Role of proflavin as a photosensitizer for the light-induced hydrogen evolution from water. *J Phys Chem* **1980,** *84* (20), 2551-2556.
3. Heelis, P. F., The photophysical and photochemical properties of flavins (isoalloxazines). *Chem Soc Rev* **1982,** *11* (1), 15-39.
4. Gross, M. A.; Reynal, A.; Durrant, J. R.; Reisner, E., Versatile photocatalytic systems for H_2 generation in water based on an efficient DuBois-type nickel catalyst. *J Am Chem Soc* **2014,** *136* (1), 356-366.
5. (a) Park, H.; Bae, E.; Lee, J. J.; Park, J.; Choi, W., Effect of the anchoring group in Ru-bipyridyl sensitizers on the photoelectrochemical behavior of dye-sensitized TiO_2 electrodes: Carboxylate versus phosphonate linkages. *J Phys Chem B* **2006,** *110* (17), 8740-8749; (b) Balzani, V.; Bergamini, G.; Marchioni, F.; Ceroni, P., Ru(II)-bipyridine complexes in supramolecular systems, devices and machines. *Coordin Chem Rev* **2006,** *250* (11-12), 1254-1266.
6. Grady, J. K.; Chasteen, N. D.; Harris, D. C., Radicals from Goods buffers. *Anal Biochem* **1988,** *173* (1), 111-115.
7. Kasuga, K.; Miyasaka, H.; Handa, M.; Dairaku, M., Photoreduction of methylviologen catalyzed by tri-sulphophthalocyaninatozinc(II) or tetra-sulphophthalocyaninatozinc(II) in aqueous-solutions. *Polyhedron* **1995,** *14* (12), 1675-1679.

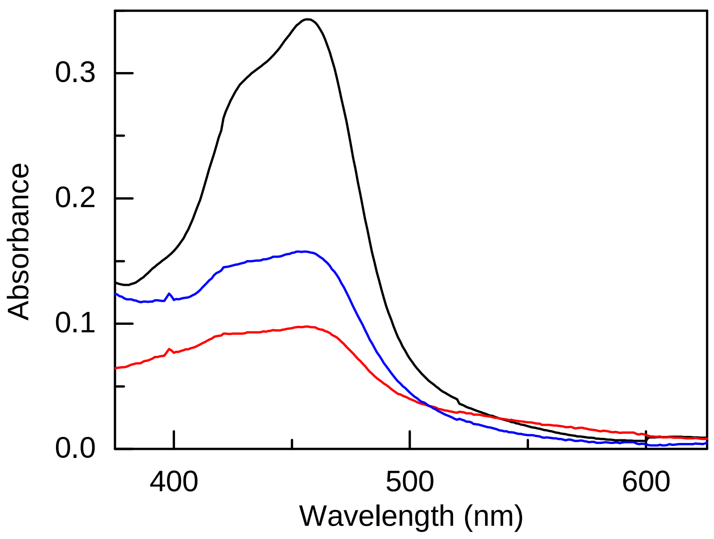

Figure S1. RuP adsorption on P25 TiO_2 particles.

Electronic absorbance of a 200 µL solution containing 33 µM RuP,150 mM MES, pH 6 (black) and the supernatant (red) recovered after 30 min incubation with TiO_2 particles (0.1 mg) followed by centrifugation to pellet the particles. The pelleted RuP coated particles were resuspended in 200 µL of 25 mM phosphate, 150 mM MES, pH 6 and after 30 min the particles were pelleted by centrifugation and the RuP content of the supernatant resolved by electronic absorbance spectroscopy (blue).

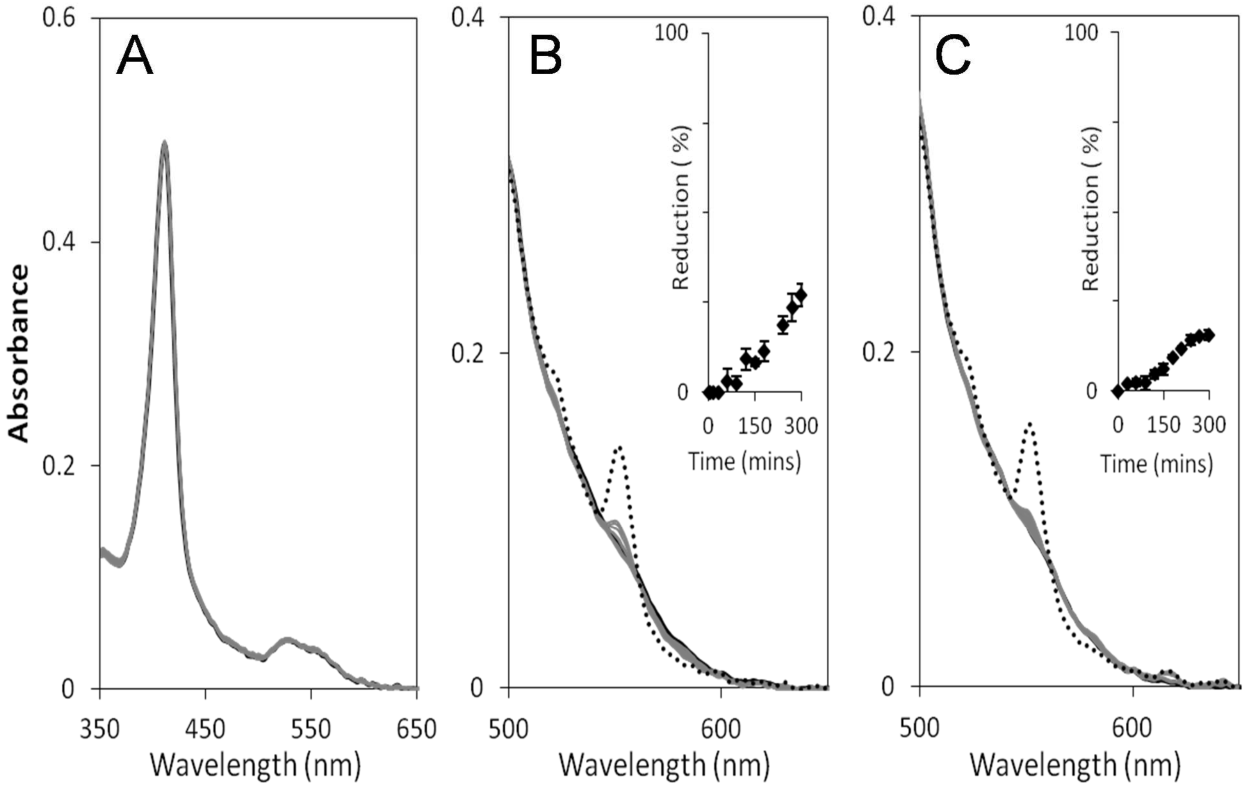

Figure S2. Electronic absorbance spectra of MtrC (0.5 μM) that assessed photo-reduction in the absence of photosensitizer (A), with 110 μM $[Ru(bpy)_3]^{2+}$ (B), and with 140 μM RuP (C).

Stirred anaerobic samples with 50 mM TEOA, 50 mM HEPES, 2 mM $CaCl_2$, 10 mM KCl, pH 7 at 20 °C (black continuous lines) were illuminated (λ > 390 nm, 400 W m^{-2}) for 5 hr (gray continuous lines). After illumination, an excess of dithionite was added to the samples containing Ru(II)-dyes (broken black lines). Path length 1 cm. Inserts: extent of heme reduction during illumination in the presence of the Ru(II)-dyes quantified through the 552 nm absorbance. Data show the average of two experiments and error bars represent the difference between the maximum and minimum values.

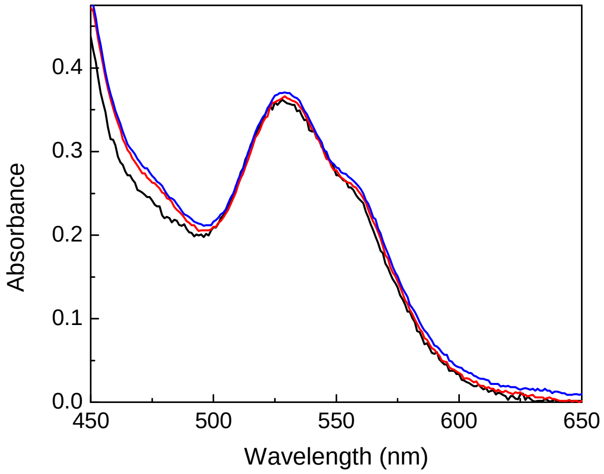

Figure S3. Formation and characterization of the flavocytochrome FMN:MtrC by electronic absorbance spectroscopy.

Flavocytochrome MtrC:FMN (3 μM) recovered by gel filtration after 30 min anaerobic incubation with reduced 1 mM glutathione, 10 μM FMN in the dark (red) and after 60 min illumination at 200 W m^{-2} (blue). Formation of the flavocytochrome is confirmed by the higher absorbance at wavelengths < 500 nm when compared to that of 3 μM MtrC (black).

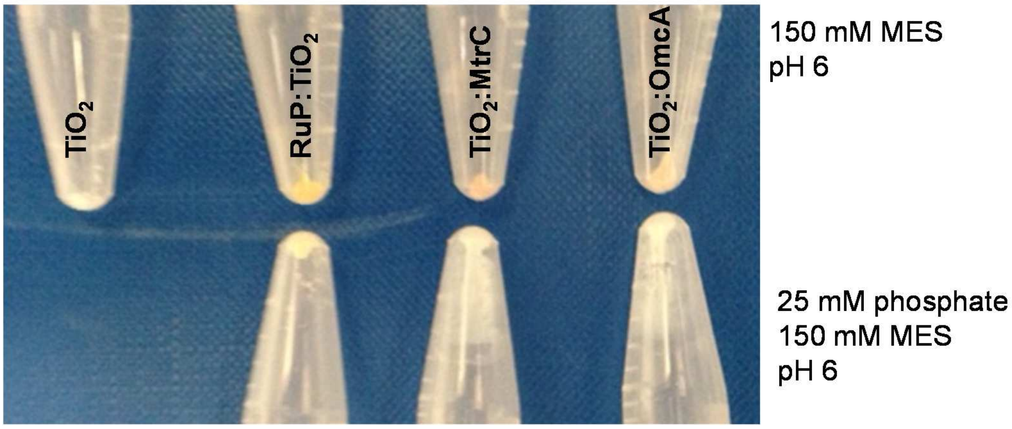

Figure S4. Adsorption of MtrC and RuP onto P25 TiO_2 particles.

Image of TiO_2 nanoparticles coated with saturating amounts of RuP, MtrC and OmcA in the presence of 150 mM MES, pH6 (upper). Adsorption of these molecules resulted in apparent color change of the particles; from white to orange with RuP and white to red with MtrC or OmcA. The RuP:TiO_2, TiO_2:MtrC and TiO_2:OmcA particles were pelleted by centrifugation (9000xg) and supernatant removed. Resuspension (30 min) of the pellets in 25 mM phosphate, 150 mM MES, pH6 resulted in the release of the RuP, MtrC and OmcA reinstating the white color of the particles when they were recovered by centrifugation (lower).

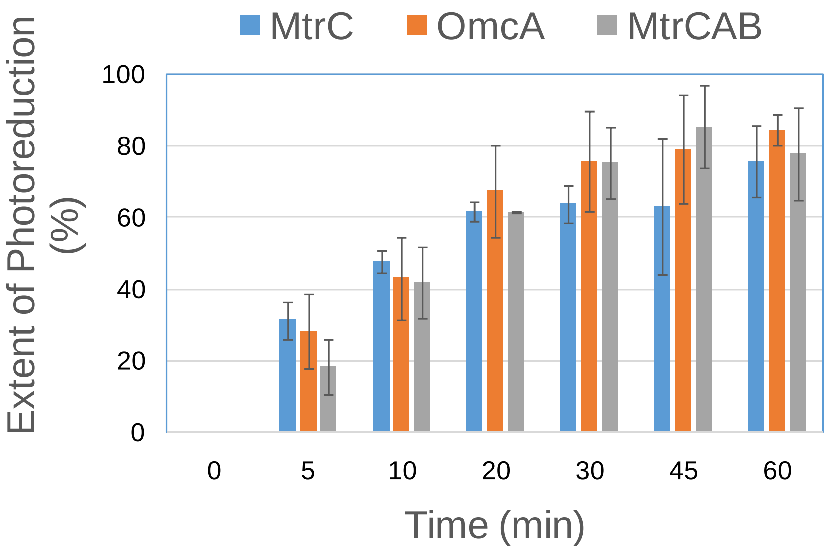

Figure S5. Time courses for RuP:TiO_2:MtrC dependent photocatalytic reduction of solutions of MtrC, OmcA and MtrCAB as indicated.

Photoreduction of stirred, anaerobic solutions of outermembrane cytochromes by RuP:TiO_2:MtrC particles (0.037 mg mL^{-1}) during 60 min illumination (λ > 390 nm, 400 W m^{-2}) at 20 ^oC. Solutions contained 0.65 µM MtrC, 0.61 µM OmcA or 0.31 µM MtrCAB as indicated in 150 mM MES, pH 6. Triton X-100 was 0.2% v/v in MtrCAB experiment. Heme reduction calculated from the absorbance at 552 nm. Data are the average and standard deviation from 3 replicates.

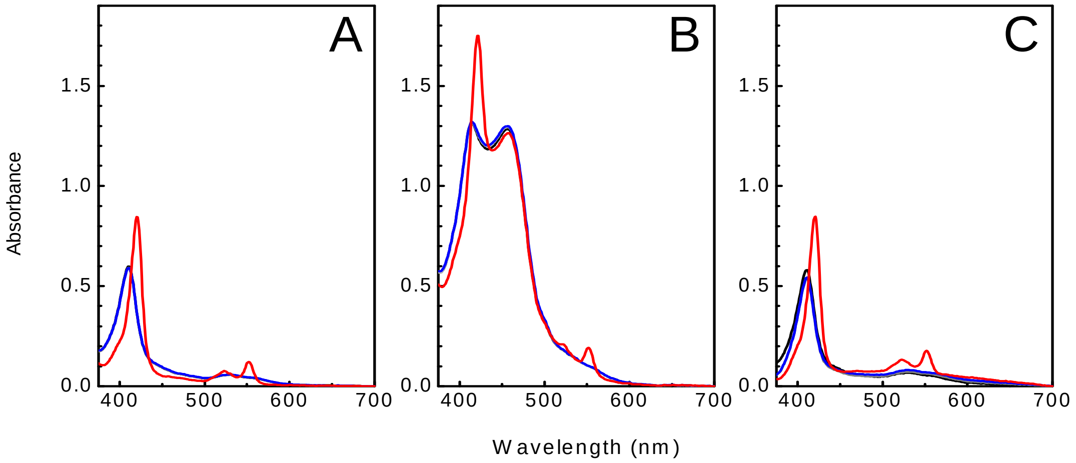

Figure S6. The impact of RuP concentration, TiO_2 and phosphate on the oxidation state of illuminated solutions of MtrC.

Electronic absorbance of 0.62 µM MtrC (black) illuminated for 1 hr (gray) and 2 hr (blue) with (A) 0.3 µM RuP, (B) 110 µM RuP or (C) 0.3 µM RuP, 0.037 mg mL^{-1} TiO_2, 25 mM phosphate followed by addition of excess sodium dithionite (red). Illumination (λ > 390 nm, 400 W m^{-2}) in stirred anaerobic solutions of 150 mM MES, pH 6 at 20 ^oC. Spectra in C) are presented after removal of the contribution due to scattering by the TiO_2 particles.

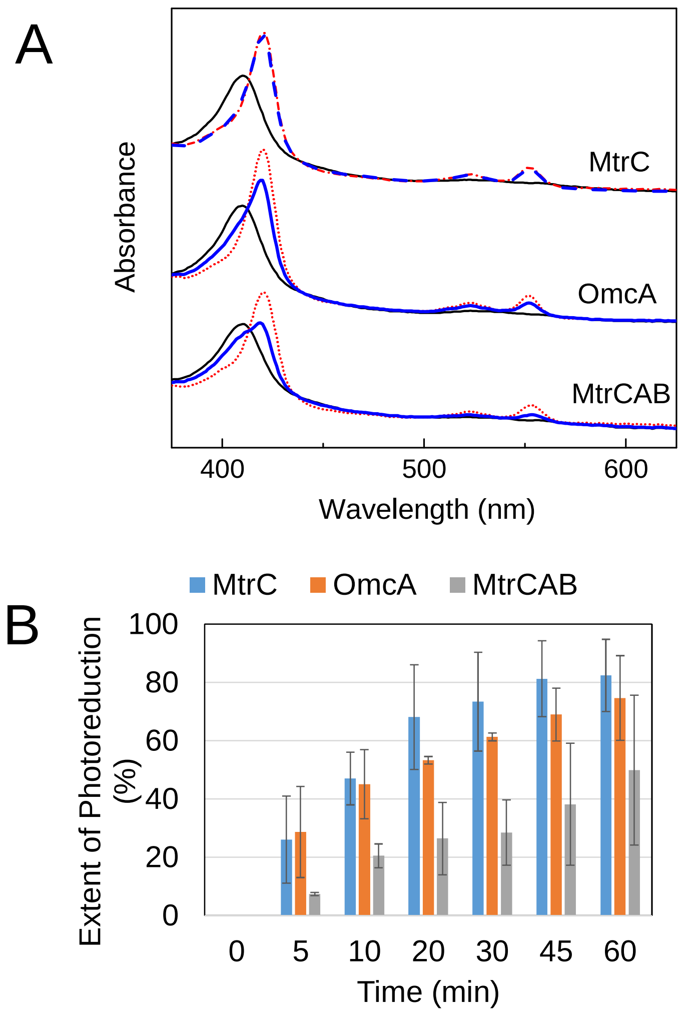

Figure S7. Photoreduction of MtrC, OmcA and MtrCAB by RuP:TiO_2:OmcA particles.

A) Electronic absorbance of 0.037 mg mL^{-1} of RuP:TiO_2:OmcA with 0.62 μM MtrC, 0.6 μM OmcA and 0.28 μM MtrCAB as indicated before (black) and after (blue) 30 min illumination (λ > 390 nm, power ≈400 W m^{-2}) followed by the addition of excess sodium dithionite (red). Spectra offset on y-axis for clarity. B) Time courses for photoreduction of the cytochrome solutions by RuP:TiO_2:OmcA. Heme reduction calculated from the absorbance at 552 nm. Data are the average of two experiments and error bars the maximum and minimum values. Experiments performed at 20 ^{o}C in anaerobic 150 mM MES, pH 6 and with Triton X-100 (0.2 % v/v) included for MtrCAB.

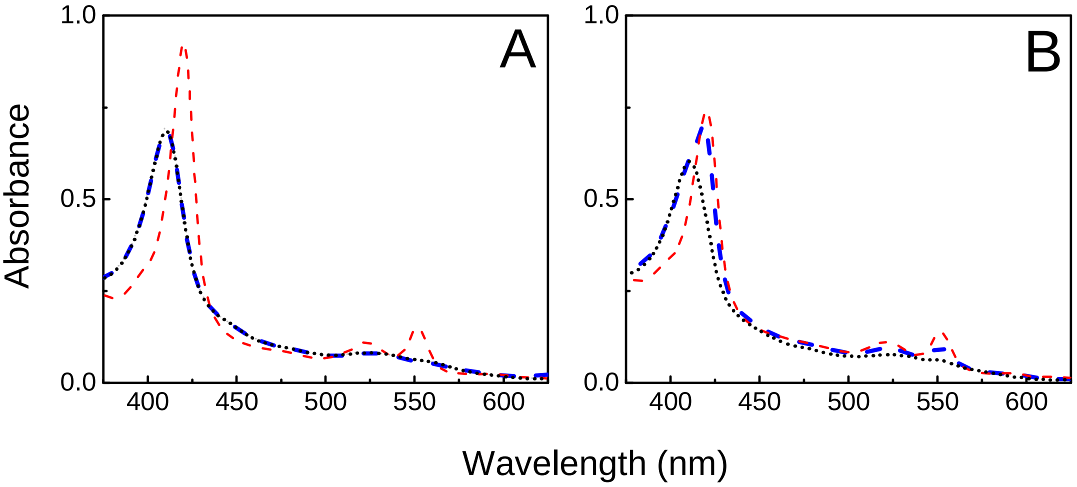

Figure S8. The impact of illumination wavelength on TiO_2:MtrC particle dependent photocatalytic reduction of an MtrC solution.

Electronic absorbance of stirred anaerobic suspensions of 0.6 µM MtrC with 0.037 mg mL^{-1} TiO_2:MtrC particles in 150 mM MES, pH 6 (gray) illuminated (400 W m^{-2}) outside the anaerobic chamber for 1 hr (blue) with light A) passed through a filter (UVK-2510, UQG Optics) to remove λ < 400 nm, or, B) direct on the sample. Subsequent addition of excess chemical reductant sodium dithionite (red) and then exposure to air (black) confirmed the protein integrity. Quartz cuvette, 1 cm path length.

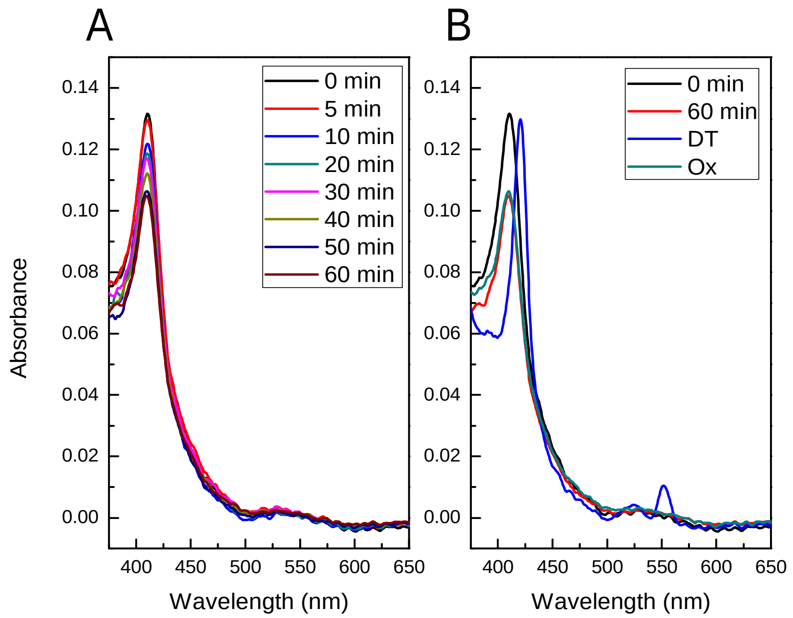

Figure S9. Electronic absorbance of TiO_2:MtrC particles illuminated (λ > 390 nm, 400 W m^{-2}) with visible light followed by addition of excess reductant dithionite and then air oxidation.

A) Electronic absorbance of a stirred, anaerobic suspension of TiO_2:MtrC particles (0.055 mg mL^{-1}) illuminated for the indicated times. B) Electronic absorbance of TiO_2:MtrC particles (0.055 mg mL^{-1}) illuminated for 0 and 60 min, followed by addition of excess chemical reductant dithionite (DT) and then oxidation in air (ox) as indicated. The spectral changes induced by the chemical oxidant and reductant after sample illumination indicate retention of the properties of functional MtrC. As a consequence the decrease in apparent absorbance below 450 nm over time in panel A) is attributed to changes in the dispersive properties of the particles most likely due to changes in aggregation. Experiments performed in 150 mM MES, pH 6 at 20 ^{o}C and spectra measured with an integrating sphere spectrophotometer.
